# Exercise-Related Oxidative Stress as Mechanism to Fight Physical Dysfunction in Neuromuscular Disorders

**DOI:** 10.3389/fphys.2020.00451

**Published:** 2020-05-20

**Authors:** Gabriele Siciliano, Lucia Chico, Annalisa Lo Gerfo, Costanza Simoncini, Erika Schirinzi, Giulia Ricci

**Affiliations:** Department of Clinical and Experimental Medicine, Neurological Clinic, University of Pisa, Pisa, Italy

**Keywords:** neuromuscular diseases, oxidative stress, physical exercise training, aerobic and anaerobic exercise, quality of life

## Abstract

Neuromuscular diseases (NMDs) are a group of often severely disabling disorders characterized by dysfunction in one of the main constituents of the motor unit, the cardinal anatomic-functional structure behind force and movement production. Irrespective of the different pathogenic mechanisms specifically underlying these disease conditions genetically determined or acquired, and the related molecular pathways involved in doing that, oxidative stress has often been shown to play a relevant role within the chain of events that induce or at least modulate the clinical manifestations of these disorders. Due to such a putative relevance of the imbalance of redox status occurring in contractile machinery and/or its neural drive in NMDs, physical exercise appears as one of the most important conditions able to positively interfere along an ideal axis, going from a deranged metabolic cell homeostasis in motor unit components to the reduced motor performance profile exhibited by the patient in everyday life. If so, it comes out that it would be important to identify a proper training program, suitable for load and type of exercise that is able to improve motor performance in adaptation and response to such a homeostatic imbalance. This review therefore analyzes the role of different exercise trainings on oxidative stress mechanisms, both in healthy and in NMDs, also including preclinical studies, to elucidate at which extent these can be useful to counteract muscle impairment associated to the disease, with the final aim of improving physical functions and quality of life of NMD patients.

## Introduction

Neuromuscular disease (NMD) is a quite broad nosographic term, which refers to different disorders either affecting the spinal cord anterior motoneuron horn cell, the peripheral nervous system, or neuromuscular junction and skeletal muscle (Iolascon et al., 2019), i.e., the components of what is termed the motor unit, an anatomic-physiological entity fundamental to realize the voluntary movement. In the case of conditions affecting the peripheral nervous system, also the sensitive pathways are involved in the pathological process, accordingly enriching clinical appearance with additional specific features.

From an etio-pathogenic point of view, NMDs are inherited or acquired conditions affecting both children and adults and ultimately leading to a motor impairment with different severity degree and time-course characteristics, sometimes with a more or less rapidly progressive evolution, sometimes with time-elapsed clinical recurrence, also life threatening, mostly implying a significant everyday life burden of disease (Silva et al., 2019). Cardiac involvement, respiratory failure, and joint contractures are among the most frequent associated features that contribute to increased disability and impaired quality of life of the patient (Mercuri and Muntoni, 2013; Morrison, 2016). Although quite homogeneous in clinical appearance, different pathogenic mechanisms (structural, metabolic, inflammatory) are involved in the process of determining cell damage in NMDs, influencing the disease course as well as its susceptibility to the available therapies. Within the chain of events that downstream follow the primary cause of the disease, several other mechanisms are thought to be important in modulating the severity of the disease; among them, a disequilibrium in the redox status, the so-called oxidative stress, has widely been considered (Bouzid et al., 2018). Such a dysfunctional toxic–metabolic state, at some extent rather common to each living cell also with protective effects in physiological contexts, can acquire a relevant pathogenic role in these conditions, in particular for its role and close relationships to the skeletal muscle contraction process and, more generally, to the physical motor activity.

Generally speaking, any type of incongruous physical activity, either physical inactivity or opposite strenuous sports, may be harmful for any organism, both in normal and illness conditions. Increased tissue peroxidative processes can be part of those dangerous scenarios through an imbalance between production of reactive oxygen species (ROS) and inactivation of antioxidant system, mostly related to a triggered mitochondrial dysfunction; this in turn leads not only to parental tissues but also to systemic, deleterious consequences that can make worse the clinical condition (Bouzid et al., 2018). NMD patients, including those with primary mitochondrial disorders as paradigmatic condition of altered redox status, are often more prone to damaging exercise, as muscle contraction itself, for different reasons, is defective and can add further damage to an already debilitated system; it turns out that they are discouraged to perform physical activity for fear of overwork weakness, rather conducing a quite sedentary lifestyle (Voorn et al., 2019). Unfortunately, however, this condition further decreases fitness and reduces individual general sense of health, as well as physical functions (Voorn et al., 2019), by favoring a deconditioning process that includes also the redox mechanism within the skeletal muscle. As a consequence of these considerations, whether or not and with which characteristics a supervised and individualized physical exercise training can be beneficial or not in NMDs is still a matter of debate, and it is even more unclear which can be its effects on muscle contractile-related redox mechanisms (Bouzid et al., 2018).

The review is aimed to focus the attention on the effects of different exercise training programs on oxidative stress, analyzing both the health status and the different pathological conditions of the neuromuscular system, with the exception of canonic mitochondrial myopathies here deliberately omitted for their peculiar significance in this context, to clarify if this may be useful to counteract muscle impairment, improving physical functions and quality of life of the affected patients.

## Materials and Methods

A literature search on PubMed was performed using the following keywords: “oxidative stress,” combined with “physical exercise,” “aerobic exercise,” “anaerobic exercise,” “resistance/strength exercise,” “training,” “healthy,” “neuromuscular diseases,” “Duchenne muscular dystrophy,” “facioscapulohumeral dystrophy,” “myotonic dystrophy,” “spinal muscular atrophy,” “amyotrophic lateral sclerosis,” and “peripheral neuropathies.” We included both *in vivo* (in animal models and humans) and, when available, *in vitro* studies, including papers up to December 2019.

## Oxidative Stress and ROS Generation

Oxidative stress describes a condition of imbalance between the production of ROS (Table 1) and the ability of the antioxidant system (Table 2) to detoxify these reactive chemical species (RCS) (Sies, 2015).

**TABLE 1:** Main reactive oxygen species.

	Symbols
**Radicals**	
Superoxide anion	O_2_⋅^–^
Hydroxyl radical	⋅OH
Peroxyl, alkoxyl radical	ROO⋅, RO⋅
Hydroperoxyl	HO_2_^–^
**Non radicals**	
Hydrogen peroxide	H_2_O_2_
Singlet oxygen	^1^O_2_
Trioxygen (Ozone)	O_3_
Hypochlorous acid	HOC1

**TABLE 2:** Main intracellular antioxidants.

	Symbols
**Enzymatic antioxidants**	
Superoxide dismutases	SODs
Catalase	CAT
Glutathione peroxidase	GPx
Glutathione reductase	GR
Glutathione-S-transferase	GST
Thioredoxin peroxidase	TrxPx
Thioredoxin reductase	TrxR
**Non enzymatic antioxidants**	
Glutathione	GSH
Glutaredoxin	Grx
Thioredoxin	Trx
Cytochrome c oxidase	
Coenzyme Q	
Ascorbic Acid	
Tocopherol	
Vitamins A, C, E	
Carotene	

Several intracellular sources of ROS have been recognized; among them, the main one is the mitochondrial respiratory chain that generates ROS as by-products of the electron transfer. Cytosolic sources, including NADPH oxidases (NOX), xanthine oxidase, lipoxygenases (LOX), cyclooxygenases (COX), and cytochrome P450 enzymes, contribute to the intracellular ROS pool. ROS are also produced by fatty acids beta-oxidation, xenobiotic components metabolism, and after the activation of phagocytosis (Mancuso et al., 2008).

Under physiological conditions, ROS are required to control biochemical processes, including cell differentiation (Vieira et al., 2011), neurogenesis (Kennedy et al., 2012), antioxidant genes expression (Allen and Tresini, 2000; Ma, 2010), as well as regulation of the immune system (Siciliano et al., 2007). However, pathological conditions in which an excessive ROS production can result in oxidative stress lead to a damage in lipids, proteins, nucleic acids, cells, and tissues, with consequent alteration of signaling pathways, inducing necrosis and apoptosis (Yan et al., 2006; Feissner et al., 2009).

In normal conditions, an efficient antioxidant defense system in the muscle fibers prevents, counteracts, or cancels the potentially damaging action of ROS on the musculoskeletal system (Cooper et al., 2002). Nevertheless, physiological levels of ROS are useful to modulate the muscle force production and to control the signaling and the genetic expression pathways in the muscle cells (Dröge, 2002).

## Physical Exercise and Oxidative Stress

The physical exercise should not be mistaken with the physical activity. The term physical activity is defined as any bodily movement, produced by skeletal muscles and that requires energy expenditure. Physical exercise is a planned, structured, repetitive, and purposeful movement to improve or maintain the physical fitness (cardiorespiratory, muscular strength, muscular endurance fitness) (Fisher-Wellman and Bloomer, 2009).

Dillard et al. (1978) for the first time observed a correlation between physical exercise and oxidative stress more than 40 years ago, demonstrating that the physical exercise can lead to an increase in lipid peroxidation. After this initial report, several studies confirmed that a prolonged and intensive exercise induces oxidative stress (Judge and Dodd, 2003; Fisher-Wellman and Bloomer, 2009; Powers et al., 2011).

It is generally assumed that an excessive physical exercise can cause oxidative stress both favoring the RCS generation, including ROS, and decreasing the antioxidant defense system. It may seemed paradoxical that, although an intense and excessive physical exercise promotes oxidative stress, a moderate and routinely exercise is associated with numerous health benefits (Powers et al., 2011), such as reducing the risk of cardiovascular, endocrine, and neuromuscular diseases (Judge and Dodd, 2003).

While an adequate exercise training is able to enhance endogenous antioxidant defense systems, reducing the harmful effect of the peroxidation processes after an intense muscle activity (Reid, 2016; Ismaeel et al., 2019), during an intense and strenuous exercise, RCS are overproduced and they are free to attack and damage any cellular component (Powers et al., 2011). It can be assumed that the physical exercise can have positive or negative effects on the oxidative stress depending on the load, specificity, and on the basal level of training (Margonis et al., 2007).

### Aerobic Exercise and Oxidative Stress

Several studies have tested the effects of the aerobic exercise (e.g., running, swimming, and cycling) on oxidative stress. An intense aerobic exercise increases oxygen consumption (VO_2_), a measure of the volume of oxygen that is used to produce adenosine triphosphate (ATP), with consequent rise in ROS production. This overproduction is not found in healthy people undergoing low exercise intensity [<50% of maximal oxygen uptake (VO_2_max)], which have a better antioxidant activity (Finaud et al., 2006).

The effect of an acute swimming protocol on the oxidative damage biomarkers was investigated in trained children (*n* = 22), who were subjected to 12 bouts of 50 m distance, at a pace corresponding to 70–75% of the maximum velocity reached, each bout separated by 1 min of rest. A significant increase in thiobarbituric-acid-reactive substances (TBARS), protein carbonyls (PC), catalase (CAT) activity, total antioxidant capacity (TAC), and oxidized glutathione (GSSG) concentration, as well as a significant decrease in reduced glutathione (GSH) concentration and GSH/GSSG ratio, were found post-exercise with respect to pre-exercise. The authors concluded that an acute swimming bout resulted in blood oxidative stress (Nikolaidis et al., 2007).

However, other studies do not confirm the evidence that oxidative stress increases with intense exercise. Inal et al. (2001) evaluated the effects of the swimming on the antioxidant status in short (100 m) and long-distance (800 m) swimmers (*n* = 10 and *n* = 9, respectively), founding that, particularly in the second ones, the antioxidant CAT, glutathione peroxidase (GPx), and GSH enzyme activity were increased.

Kouvelioti et al. (2019) observed that oxidative stress biomarkers (TBARS and PC) varied similarly after a running or cycling training. Specifically, 20 healthy men (22.3 ± 2.3 years) performed two high-intensity interval exercise trials (crossover design), running on treadmill and cycling on cycle ergometer. Trials consisted of eight running or cycling intervals (lasting 1 min) at ≥90% of the maximum heart rate (HR_max_), separated by of passive recovery intervals (1 min). The duration but not the type of the exercise influenced the level of oxidative stress markers; in particular, TBARS and PC did not change from pre- to 5 min post-exercise but significantly decreased from 5 min to 24 and 48 h post-exercise (Kouvelioti et al., 2019).

This could be explained based on the time required to activate the biological pathways underlying the cellular redox state after an aerobic exercise. It is well known that physical exercise induces, in the immediate, an increase in ROS; appreciable positive changes in redox status cannot occur during, or immediately after, the exercise but are required several hours (e.g., 9, 24, 48 h) after the exercise end. It can be speculated that this timeframe is compatible and is required for antioxidant gene transcription activation, the messenger RNA (mRNA) maturation, and its translation into protein, [i.e., superoxide dismutase (SOD), CAT, GPx]. Exercise induces a pleiotropic adaptive response in skeletal muscle, through the activation of transcription factors (e.g., peroxisome proliferator-activated receptor g coactivator 1a, PGC-1a) that regulate mitochondrial biogenesis and activate the transcription of antioxidant enzymes (Pasquinelli et al., 2016). Thus, if the aerobic training is performed for a long time, not only for a limited period (a few weeks), its positive effects on oxidative stress can persist over time, since it keeps the antioxidant enzymatic machinery active.

Nonetheless, the contrasting results of the literature can be explained by different antioxidant nutritional status or by different intensity, duration and frequency of the training, type of the exercise (Finaud et al., 2006), previous training experience, inclusion of an untrained control group, number of participants, as well as anthropometric parameters such as body mass index (BMI), age, and sex (Celik et al., 2019). It is possible that sex may influence the degree of oxidative stress; women can be less susceptible to oxidative stress than men considering the antioxidant properties of estrogens, especially during periods when estrogens levels are high (e.g., ovulation) (Bloomer et al., 2009). Nevertheless, only few studies regarding the aerobic exercise-related oxidative stress, in healthy human, evaluated and found differences among sexes in oxidative stress biomarkers (Bloomer and Fisher-Wellman, 2008; Kabasakalis et al., 2014; Souglis et al., 2018; Celik et al., 2019).

### Anaerobic Exercise and Oxidative Stress

It is known that the anaerobic exercise, such as sprints, intermittent running, jumps, or resistance exercises, is a source of free radicals, produced by xanthine oxidase (XO), an enzyme that generates ROS during the ischemia reperfusion (Heunks et al., 1999). XO, in the presence of hypoxanthine or xanthine, reduces molecular oxygen in superoxide anion (O_2_-) and hydrogen peroxide (H_2_O_2_), which, in turn, is reduced into hydroxyl radical (OH), inducing oxidative damage (Heunks et al., 1999). ROS are also produced during myocyte contraction by NADPH oxidase and nitric oxide synthase activation (Powers and Jackson, 2008). Elevated lactate levels, as well as an alteration of the oxidative status, assayed by an increase in the lipid peroxide concentration were found after anaerobic exercise (Powers and Jackson, 2008). In addition, gender differences need to be considered when anaerobic activity is evaluated. The myocyte metabolic activity and mass and histology of the skeletal muscles are different in men with respect to women (Janssen et al., 2000; Esbjornsson-Liljedahl et al., 2002). Men show higher peak of anaerobic power that is correlated positively with post-exercise oxidative stress that, in turn, is influenced by the type II muscle-fiber composition. In fact, the type II fibers have intrinsic properties that favor ROS production, with a consequent increase of PC (Quindry et al., 2011).

Studies evaluating enzymatic antioxidant status have not yet provided clear evidence that anaerobic exercise prevents the trigger of oxidative stress. A study with healthy male volunteers (*n* = 8) demonstrated that short-term supramaximal anaerobic exercises (the 30 s Wingate test) decreased the SOD activity, suggesting that this type of exercise induces oxidative stress, although no changes in GPx activity were detected (Groussard et al., 2003). Conversely, Berzosa et al. (2011), in healthy untrained male subjects (*n* = 34) undergoing to cycloergometric tests, including maximal and submaximal episodes, found an increase in CAT, GPx, glutathione reductase (GR), and SOD enzyme activities in plasma after both maximal and submaximal exercise periods.

A study by Wiecek et al. (2015) aimed to evaluate the changes in non-enzymatic antioxidants in 20 healthy individuals (both man and women) undergoing an anaerobic exercise (20 s bicycle sprint). In both sexes, total oxidative status (TOS), TAC, TOS/TAC, vitamin A, vitamin E, vitamin C, uric acid, and GSH concentrations raised after exercise, suggesting that also the anaerobic exercise may play a beneficial role because of its ability to increase the antioxidant defenses.

The effect of a resistance training (RT) on oxidative stress biomarkers depends substantially on the type of training carried out. A moderate-intensity RT, rather than a maximal exercise, is able to improve adaptive responses to oxidative stress by upregulation of antioxidant defenses, limiting the formation of free radicals and preserving the reducing power after a moderate-intensity and low-frequency training (Jürgenson et al., 2019).

When an anaerobic training protocol is proposed, several confounding factors should be taken into consideration, e.g., type, intensity of exercise performed, sex, and training status of the participants.

## Oxidative Stress and Exercise Training in Neuromuscular Disorders

It is widely known that oxidative stress is linked to the pathogenesis of NMDs (Wehling et al., 2001). Several studies were conducted both in animal models and humans affected by NMD, covering a wide spectrum of pathologies: from muscular dystrophies to spinal muscular atrophy (SMA), amyotrophic lateral sclerosis (ALS), and peripheral neuropathies (PN) (Cassereau et al., 2020).

Despite that oxidative stress is involved in NMDs, and exercise induces ROS production, some types of training increase mRNA levels and the expression of metabolic muscle proteins. In particular, a moderate and regular physical exercise has been suggested as non-pharmacological treatment for the NMDs (Sjøgaard et al., 2013).

### Muscular Dystrophies

#### Duchenne Muscular Dystrophy

Oxidative stress has been proposed as a secondary effect in the Duchenne muscular dystrophy (DMD) (Serra et al., 2018). An alteration of the oxidative balance was detected both in *mdx* mice and in muscle biopsies or blood from DMD patients. TBARS, a by-product of lipid peroxidation, was found higher in *mdx* mice *versus* controls muscles (Ragusa et al., 1997), and also protein oxidation levels were reported in *mdx* mice (Dudley et al., 2006). Similarly, TBARS (Kar and Pearson, 1979), carbonyl-proteins (Petrillo et al., 2017), and 8-OHdG (Rodriguez and Tarnopolsky, 2003) were found elevated in the muscle and blood of DMD patients *versus* healthy controls. While the literature data agree on the increased oxidative damage DNA, proteins, and lipids, data related to antioxidant status in DMD are conflicting. Some studies reported higher levels of SOD-2 and CAT, while decreased SOD-1 activity was found in *mdx* mice (Ragusa et al., 1997). In addition, DMD patients showed an increased muscle enzymatic activity of CAT and GR than controls, while SOD activity was not altered (Kar and Pearson, 1979). Another study found decreased levels of GSH with an increased GSSG/GSH ratio, and a greater activity of GPx and GR in *mdx* mice muscles (Dudley et al., 2006). Recently, Petrillo et al. (2017), analyzing the antioxidant status in DMD patients with respect to healthy subjects, observed a decrease in GSH levels and GPx activity, as well as an imbalance of GSSG/GSH ratio in muscle and plasma.

An eccentric exercise can damage the contractile and cytoskeletal components of the muscle fibers; it may have dangerous effects resulting in muscle deterioration (Markert et al., 2011), In addition, high-resistance strength training is not recommended in DMD, but submaximal aerobic exercises can be helpful to improve the muscle functions (Yiu and Kornberg, 2015).

##### Exercise-related oxidative stress in DMD animal models

Voluntary running exercise for 7 weeks performed by *mdx* mice improved citrate synthase and succinate dehydrogenase activities, both markers of muscle aerobic capacity, and it enhanced the expression of aerobic metabolism genes to levels similar or higher than those observed in healthy mice (Hulmi et al., 2013).

Sedentary *mdx* mice, with downregulated levels of SOD-1, when subjected to low-intensity endurance exercises (run on a motorized rotarod for 5 days/week for 6 weeks), showed restored normal levels of SOD-1, suggesting that specific programs of training could contribute to a significant recovery of the damaged muscle, counteracting oxidative stress and reducing the muscle degeneration (Fontana et al., 2015).

In addition, Hyzewicz et al. (2015) agree that low-intensity training may have a beneficial effect on dystrophic muscle in *mdx* mice. In fact, a voluntary swimming training for 30 min, 4 days/week for 4 weeks, improved the expression of proteins involved in energy metabolism and in muscle contraction, as well as decreased oxidized proteins (Hyzewicz et al., 2015). Low-intensity treadmill training (9 m/min, 30 min/day, three times/week, for 8 weeks) reduced collagen deposition, enhancing both enzymatic and total antioxidant capacity (Fernandes et al., 2019).

These results could be useful to design correct exercise training protocols for DMD patients.

##### Clinical trials in DMD patients

Few human studies have focused on exercise training in DMD. An important purpose in DMD is delaying the loss of the motor functions, which negatively impacts DMD patients’ life. As asserted by Jansen et al. (2010, 2013), physical training could be effective to limit the deterioration of muscle tissue. The authors examined the effects of a low-intensity physical training (5 days/week for 24 weeks), on muscle endurance and functional abilities, in 30 young DMD patients (18 ambulant and 12 wheelchair dependent; mean age, 10.5 ± 2.6 years), divided into two groups: a trained group (*n* = 17) and a control group (*n* = 13) of untrained patients. The intervention group trained their limbs in 30 min sessions (15 min for arms and 15 min for legs) by a bicycle training equipment used actively or with electrical motor support, depending on their physical limitations (Jansen et al., 2010). In the trained group, the total Motor Function Measure (MFM) score remained stable, whereas it significantly decreased in the control group. No significant differences were found for the Assisted 6-Min Bicycle Test (A6MCT). A low-intensity cycling training could be beneficial, feasible, and safe for both ambulant and wheelchair-dependent children, perhaps delaying the functional muscle deterioration due to disuse in DMD patient (Jansen et al., 2013).

A clinical trial with adult Becker muscular dystrophy patients (*n* = 11) subjected to an aerobic and moderate-intensity training (training session of 50, 30 min on a stationary cycle ergometer at 65% of VO_2max_) confirmed the effectiveness of the exercise protocol with persisting positive effects after 3 months of follow-up. Six patients continued the same training protocol for the next 12 months, highlighting the importance of the proposed rehabilitation exercises in dystrophinopathies (Sveen et al., 2008).

Alemdaroğlu et al. (2015) investigated the effects of exercise programs for strengthening range of motion with an arm ergometer, in early-stage DMD patients. Ambulation scores, endurance, arm functions, and proximal muscle strength were improved after 8 weeks of training.

#### Facioscapulohumeral Dystrophy

The involvement of oxidative stress in facioscapulohumeral dystrophy (FSHD) is predominantly supported by *in vitro* studies; myoblasts from FSHD patients are susceptible to oxidative insults early during FSHD myogenesis (Winokur et al., 2003), contributing to an aberrant differentiation of myoblasts (Dmitriev et al., 2016). This condition can be reversed to a normal state by treatment with antioxidants, reducing the oxidative damage and the morphological defects during the myogenic differentiation (Dmitriev et al., 2016).

Using myocytes differentiated from induced pluripotent stem cells (iPSCs) derived from FSHD patients, Sasaki-Honda and collaborators (Sasaki-Honda et al., 2018) demonstrated that oxidative stress increased DUX4 expression by a DNA damage response signaling to the opened FSHD 4q35 region. Using isogenic controls of FSHD2 iPSCs in order to correct the SMCHD1 mutation, basal DUX4 expression was suppressed and heterochromatic markers at 4q35 were partially recovered, suggesting that oxidative stress could represent a risk factor in FSHD progression (Sasaki-Honda et al., 2018).

Abnormal mitochondrial functions and increased levels of oxidative stress biomarkers (e.g., lipid peroxidation, PC, and lipofuscin accumulation) were detected in FSHD compared to healthy muscles (Turki et al., 2012).

In patients with FSHD, some strategies, including antioxidant supplementation and physical exercise training, could be considered to improve the muscle function performance compromised by oxidative stress.

A clinical randomized controlled trial with FSHD patients reported, after antioxidant supplementation with vitamin C, vitamin E, zinc gluconate, and selenium (once a day, for 17 weeks), an enhanced muscle function evaluated by 2-min walking test (2-MWT), maximal voluntary contraction (MVC), and endurance limit time. MVC is the expression of an isometric muscle contraction expressed as the percent of strength for the design person. Non-enzymatic antioxidant and lipid peroxidation biomarkers were also improved in treated patients *versus* placebo group, concluding that the supplementation of antioxidant substances can really help to improve physical performance by enhancing antioxidant defenses and reducing oxidative stress (Passerieux et al., 2015).

How exercise training can be useful in FSHD has been observed by Voet et al. (2014), who subjected 28 FSHD patients to an aerobic exercise training, lasting 16 weeks, which consisted of three sessions/week of cycling exercises, for 30 min with additional warming-up (5 min) and cooling-down (3 min) periods. During the training period, cardiovascular load was monitored with a heart rate belt and watch and adjusted to the individual participant’s level, reaching an increase of 50–65% in heart rate reserve (HRR), normally calculated as the difference between the maximum heart rate (HR_max_) and resting heart rate (HR_rest_). After training, the patients showed a reduction in fatigue, assessed by the fatigue subscale of the Checklist Individual Strength (CIS fatigue), and the beneficial effects persisted over time, at 16 and 28 weeks of follow-up.

One study, aimed to assess in FSHD the efficacy of 6 months of home-based exercise training program (cycling three times/week, for 35 min), consisting in a combination of strength, high-intensity interval, and low-intensity aerobic exercise, has shown improvement on VO_2max_, MVC, and muscle endurance without the evidence of muscle tissue damage (Bankolé et al., 2019).

Studies evaluating the effect of training on muscle performance did not find evidence of enhancing by isometric strength testing, supporting the idea that strength-training exercise is probably ineffective for significantly improving muscle strength in FSHD (Tawil et al., 2015). Therefore, a stationary bicycle rather than a treadmill should be recommended for patients with leg weakness (Tawil et al., 2015). Although no data suggest that strength training is detrimental in FSHD and considering these evidence, patients should be encouraged to perform low-intensity aerobic exercises (Tawil et al., 2015).

#### Myotonic Dystrophy

Type 1 myotonic dystrophy (DM1) is a neuromuscular disease characterized by multisystemic involvements, in which oxidative stress may be associated with muscular signs of disease, contributing to muscle atrophy (Toscano et al., 2005; Miljević et al., 2010). An increase in protein oxidation (Siciliano et al., 2005) that correlates with extramuscular signs of the disease, as well as an increase in lipid peroxidation (Kumar et al., 2014), was found in plasma of patients with DM1. In addition, antioxidant status is compromised, as demonstrated by lower levels of GPx, glutathione S-transferase (Kumar et al., 2014), SOD, and CAT (Nikolić-Kokić et al., 2016) detected in DM1 patients than in controls.

The effects of exercise and physical training to counteract the progressive loss of maximal muscle strength and muscle wasting have been well documented (Roussel et al., 2019).

In DM1 patients, strength testing (single session exercise, at 50% of MVC until exhaustion) showed lower maximal strength and longer recovery time compared to healthy controls, which are in line with muscle weakness and myotonic phenomenon (Esposito et al., 2017). In a 12-week aerobic training protocol on a cycle ergometer, in which each training session consisted of 5 min of warm-up and 30 min of exercise at 65% of the VO_2max_, positive effects on the aerobic capacity, fitness performance, and self-reported improvement in daily activities were observed. In addition, a post-training histological analysis showed a muscle fiber remodeling, with an increase in types I and IIa fiber diameter, with respect to the basal condition, whereas fiber density was not changed (Orngreen et al., 2005).

Nevertheless, according to a single-case study design, five DM1 patients underwent a hand exercise program focused on endurance training at low resistance, with a duration of ∼45 min. The training sessions, three times/week, for 12 weeks, included isolated sets of different exercises in 3 or 5 repetitions and mass sets of movements with a Theraputty in 10 or 15 repetitions, followed by a stretching session. A significant increase in muscle force and an improved fine motor control were reported, whereas no statistical significant difference was found in grip force. A higher occupational performance was self-reported when asked (Aldehag et al., 2005).

These data have been confirmed in patients (*n* = 20) subjected to a composite rehabilitation program consisting of 15 sessions spread out over a 6-week period. Stretching exercises for muscle stiffness and strengthening, balance, and endurance training were performed. In particular, the endurance training consisted of walking on a treadmill (a 20 min session, at 60% of the VO_2max_).

The results on endurance were not conclusive, probably because the proposed endurance training program was insufficient to improve the walking perimeter, while muscle strength and locomotor and posture locomotion improved (Missaoui et al., 2010).

Type 1 myotonic dystrophy patients (*n* = 20) underwent an aerobic exercise protocol with a hand grip, consisting of three sets of intermittent fatiguing contractions, at incremental working load up to 60% of MVC, lasting 1 min, each separated by 1 min of rest. The lactate acid levels increased at 60% of MVC, while oxidative stress markers did not differ from baseline (Baldanzi et al., 2017).

#### Spinal Muscular Atrophy

An abnormal deposition of 4-hydroxy-2-non-enal-modified protein (4-HNE), a product of membrane lipid oxidation, was observed at immunohistochemical staining in the spinal motor neurons of patients with SMA type 1. In addition, an immunoreactivity for 8-hydroxy-2-deoxyguanosine (8-OHdG), a marker of oxidative DNA damage, was observed in SMA (Hayashi et al., 2002). Mano et al. (2012) subsequently confirmed that urinary 8-OHdG levels in SMA patients were higher compared to controls and that they were correlated with the motor function scores and the disease duration.

In SMA, physical exercise training might improve muscle and cardiorespiratory function. As described by Bartels et al. (2019), aerobic exercise training optimized the aerobic capacity, counteracting the muscle deterioration that occurs secondarily to motor neuron loss and inactivity.

Spinal muscular atrophy type 3 patients, because of a milder progression of the disease, could represent an ideal target population for applying strengthening and endurance exercise protocols. Nevertheless, no conclusive data are available regarding the protective role of the physical exercise in this type of patients (Madsen et al., 2015).

Six SMA type 3 patients subjected to 3 months of training, two to four sessions for a week, on a cycle ergometer for 30 min at 60–70% of the VO_2max_, and a control group of nine sedentary subjects were compared, and it was found that the patients’ oxidative capacity and the endurance improved after the training. On the contrary, a significant fatigue was reported, confirming that SMA patients are susceptible to exercise-induced fatigue (Bartels et al., 2019).

Other studies found that, in SMA patients trained with the same exercise protocol, which differed only in the duration (6 *versus* 12 months in the two patient groups), no significant differences were reported regarding fatigue (Montes et al., 2015).

#### Amyotrophic Lateral Sclerosis

Amyotrophic Lateral Sclerosis is considered a multisystem and multifactorial pathology in which several mechanisms play important roles in the development and progression of the disease (Cozzolino et al., 2008).

The etiology and pathogenesis of the neuronal apoptosis in ALS are currently largely unknown. Among the several hypotheses, oxidative stress could be a driver in the neurodegenerative processes (Cozzolino et al., 2012; Gagliardi et al., 2012; Mao et al., 2012), including ALS. High levels of oxidative stress biomarkers, such as 4-HNE (Beal et al., 1997; LoGerfo et al., 2014), mitochondrial dysfunction (Shaw et al., 1995; Cutler et al., 2002), and, on the contrary, a downregulation of glutathione S-transferases (GSTs) (Curti et al., 1996; Simpson et al., 2004), peroxiredoxins (Kong and Xu, 1998), and nuclear factor erythroid 2-related factor 2 (Nrf2) (Wiedemann et al., 2002; Kato et al., 2005; Kirby et al., 2005; Usarek et al., 2005) were observed in ALS patients (Barber et al., 2006; Sarlette et al., 2008; Strong, 2010). Furthermore, Nrf2 activators have been shown to protect against oxidative stress and cell death induced by SOD1-mutant protein (Rossi et al., 2012). The *NRF2* overexpression in glial cells increases the resistance to oxidative stress and, by the increase in GSH levels, the ability of the motor neurons to neutralize the toxic effects caused by SOD1-mutant protein (Gitcho et al., 2008). In addition, a reduction in Nrf2 protein was found in ALS patients than in controls (Vargas et al., 2008; Kanno et al., 2012; Mead et al., 2013).

In the last decade, genome-wide association studies identified two genes associated with sporadic and non-SOD1 familial ALS: RNA/DNA-binding proteins, 43 kDa transactive response (TAR) DNA-binding protein (TDP-43), and fused in sarcoma/translocated in liposarcoma (FUS/TLS) (Kato et al., 2000; Neumann et al., 2006; Guo et al., 2013; Milani et al., 2013). Both TDP-43 and FUS, which predominantly nuclear proteins involved in RNA metabolism, are observed as aggregates in the cytosol of ALS neurons (Mackenzie et al., 2007).

In addition, *in vitro* studies, NSC34 motor neuronal cell lines, expressing TDP-43 mutants, exhibited shortened neuritis and higher oxidative stress. These effects were reversed by the UPS inhibitor MG132, but not by the Nrf2 activator sulforaphane (Vance et al., 2009; Vargas et al., 2013). This evidence was attributed to an increase in heme oxygenase 1 (HO-1), following MG132 treatment, apparently independent from Nrf2 activation. While the protective role of Nrf2 in SOD1-related toxicity is clear, the effect on other ALS-associated genes (e.g., TDP-43 and FUS) needs to be clarified (Vance et al., 2009; Vargas et al., 2013).

To date, few studies evaluated the correlation between oxidative stress and exercise in ALS. Among them, Flis et al. (2018) demonstrated that a swimming training is able to extend the lifespan of ALS G93A mice. The transgenic mice performed an exercise protocol that started at the presymptomatic stage (70 days of age) and ended when the mice were 115 days of age. The protocol consisted of a swim for 30 min, in 30°C water, in a swimming pool with an adjustable flow of water (maximum rate of 5 L/min), for five times/week. On the 105th day of life, the frequency of training was reduced to three times/week, and the daily swimming time (maximum of 30 min), and water flow (maximum of 5 L/min) was set individually, according to the abilities of the ALS mice. This exercise protocol could have had beneficial effects on the lifespan of ALS mice, probably because it may induce changes in oxidative stress and bioenergetics. In particular, higher levels of CAT activity were observed in the trained ALS mice than in sedentary ALS group of mice, although higher thiols (SH) oxidation was found both in the ALS sedentary group and in the trained group. Moreover, the COX activity and the RCR increased in the trained group *versus* the sedentary group, evaluated in the skeletal muscle of ALS mice. Additionally, also markers of lipid peroxidation were measured in the crude mitochondrial fraction isolated from the skeletal muscle, and it was found that there was a reduction in lipid peroxidation in the mitochondria of the ALS-trained *versus* ALS no-trained groups. These protective changes induced by swimming could be explained as the result of a reduction in the cholesterol content and an increase in the caveolin-1 protein level in the crude mitochondria of the trained ALS mice skeletal muscle.

These positive results could encourage swimming-based training programs that may be helpful in ALS patients. Swimming exercises produce physically and psychologically positive effects, as well as neuroprotective effects improving the motor functions as observed in ALS mice (Just-Borràs et al., 2019), suggesting a possible future use of well-structured swimming-based training protocols also in ALS patients. ALS mice subjected to swimming (in an adjustable-flow swimming pool) or to running (on a treadmill) protocols (for 30 min a day, 5 days/week, for 12 weeks) showed delayed spinal motoneuron death and preserved the motoneurons with large soma area. In contrast, a substantial motoneuron loss and an increased proportion of motoneurons with small soma area were observed after running-based training (Just-Borràs et al., 2019). Running is a high-impact exercise, which recruits only small motoneurons and generates more oxidative stress than swimming, which is a low-impact exercise that recruits both small and big motoneurons, probably mediated by exacerbated fast-to-slow functional transition by running, while swimming preserves the normal muscle phenotype (Just-Borràs et al., 2019). Therefore, it can be assumed that, although both protocols induce changes in the proportion of motoneurons, swimming mitigates the vigorous loss of big and fast motoneurons unlike running (Just-Borràs et al., 2019).

Although preclinical studies (Flis et al., 2018; Just-Borràs et al., 2019) suggest that swimming and aquatic training could be very encouraging exercise interventions in ALS, clinical studies are needed to translate these mouse exercise results to humans, considering also that personalized aquatic training programs will be needed.

To our knowledge, only two studies that evaluated the exercise-induced oxidative stress remodulation in ALS have been conducted in humans (Pasquinelli et al., 2016; Chico et al., 2018). In both studies, the exercise protocol, on the forearm muscles with a myometer, consisted in several steps in which the contractile force increased incrementally at 30, 50, and 70% of the MVC, each step including 1 min of intermittent contractions (∼1/s) and 2 min of rest.

In the first of these two studies Pasquinelli et al. (2016) found that the plasma levels of oxidative protein products (AOPPs), ferric-reducing ability (FRAP), and total thiol groups (t-SH) remained stable during this short-lasting exercise protocol, suggesting a more delayed, if any, exercise-related kinetic curve for these redox biomarkers. On the contrary, the levels of lactate during each step of the exercise protocol increased, although no difference was observed when comparing the curves of ALS patients and healthy subjects. Interestingly, patients genotyped for the Gly482Ser PGC-1α gene polymorphism (during exercise, muscle contraction causes PCG-1α protein activation, increases mitochondrial biogenesis and oxidative capacity, leading to fast-to-slow muscle fiber conversion) showed a different response on oxidative stress-related biomarkers, according to their genotype. In particular, patients harboring the Ser428Ser genotype showed, during exercise, higher levels of AOPP (at 50% of MVC and at recovery) and lactate (at 30 and 50%) compared to Gly482Gly patients, highlighting a possible role of PGC-1α in exercise-related oxidative stress.

In the second study, Chico et al. (2018) evaluated, over a period of 6-month trial and compared to clinical scores, the exercise-related effects, on the same redox parameters, of the oral administration of a curcumin-based compound (600 mg/day, Brainoil), a natural antioxidant nutraceutical, in ALS patients. Patients were randomized into two groups: group A (*n* = 24) received placebo for 3 months, then Brainoil for the following 3 months; group B (*n* = 18) took Brainoil for 6 months. Evaluations were conducted at basal (T0), after 3 months of double-blinded Brainoil/placebo treatment (T1), and after 3 months of open-label phase (T2). After curcumin administration, an improvement in oxidative stress biomarkers evaluated during the incremental forearm exercise test was observed. In particular, the intragroup analysis showed a significant reduction in AOPPs at 30 and 50% of the MVC at T2 > T0 and T2 > T1 in ALS patients belonging to group B who consumed Brainoil for 6 months. Moreover, at T2, group B showed, at 50% of MVC, lower AOPP levels than group A. In group A, a significant reduction in FRAP values both at 70% of MVC and at recovery was observed at T1 > T0, suggesting that during placebo administration, there was worsening of FRAP. At T2, when Brainoil was introduced in the diet of group A, a FRAP restore was observed and rising FRAP levels, at different steps of the exercise-related curve, comparable to those of T0. Finally, in group B, relative lactate levels decreased at 50% of MVC at T1 > T0. Comparing groups A and B, there were no significant differences both in absolute and relative lactate levels, at T0 and T2, while at T1 in group A, lactate absolute values increased with respect to group B at 50 and 70% of MVC. The evaluated biomarkers improved in the treated patients group, suggesting that curcumin oral administration could have a protective effect during muscle exercise (Chico et al., 2018).

However, conclusive data regarding the effect of oxidative stress on physical performance in ALS patients, and conversely, if the muscle activity can modify the redox status, are still lacking. While some studies highlight the positive effect of physical exercise on the quality of life in ALS patients (Lehman et al., 2012), others argue that exercise has a harmful effect on muscle function (Lunetta et al., 2016).

In previous studies, strenuous exercise was associated with higher ALS incidence, as observed in athletes (Strickland et al., 1996; Scarmeas et al., 2002; Gotkine et al., 2014) and professional football and American football players (Chio et al., 2005; Lehman et al., 2012). Probably, an intense and prolonged exercise could induce both a massive increase in ROS and calcium concentration, with consequent motoneurons degeneration (Harwood et al., 2009), as observed in ALS patients in an extra 10 kJ/kg/day of physical activity, equivalent approximately to 45 min of brisk walking. This was consistently associated with an increased risk of ALS, with the strongest association observed for adulthood exercise-related physical exercise (Harwood et al., 2016). Furthermore, neurons, more than other cells, are particularly susceptible to oxidative stress due to high oxygen demand and low amount of antioxidants (Halliwell, 2006). Nevertheless, epidemiological data on the association between sport and ALS are contrasting. A study conducted by Belli and Vanacore (2005) observed a high risk for ALS among Italian soccer players. In this scenario, also drugs and food supplements intake should be discussed (Luna et al., 2017), considering that they are used to enhance sports performance. A high consumption of dietary supplements containing branched chain amino acids (BCAAs) and a chronic misuse of anti-inflammatory drugs could play an important role in the etio-pathogenesis of ALS among susceptible athletes (Belli and Vanacore, 2005). In fact, a dangerous interaction between genetic susceptibility and environmental factors can occur (Roche et al., 2012), but no data regarding doping and ALS are given in the literature.

However, it is important to emphasize that physical activity promotes the production of neurotrophic factors, as observed in adult rats subjected to exercise training (5 days, 30 min/day, at a speed of 27 m/min, at a 3% incline using a motor-driven treadmill belt) (Gomez-Pinilla et al., 2001), with a protective effect on spinal cells, synaptic remodeling, and hyperinnervation of neuromuscular junctions (Nguyen et al., 1998; Wehrwein et al., 2002). Furthermore, it has been observed that endurance exercise is able to reactivate pathways interrupted by the disease, as it triggers remodeling mechanisms in the muscle fiber cells. In particular, endurance exercises protect skeletal muscle against the excessive activation of autophagy and ubiquitin–proteasome system, act by upregulating mitochondrial metabolism and fiber-type transformation, and stimulate the mitochondrial biogenesis and the expression of mitochondrial respiration genes (Ferraro et al., 2014). Considering these conflicting data, it can be assumed that there could not be a direct association between sport activity and the onset of ALS.

It is well known that regular and moderate physical activity protects neuronal cells from ischemic damage as demonstrated in hippocampal tissues of ALS mouse models (Tsitkanou et al., 2019). A study conducted by Pinto et al. (1999) showed that a moderate-intensity training on a treadmill (12 months), coupled with the use of a non-invasive ventilator, reduced the percentage of respiratory deterioration compared to an untrained group.

Similar results were obtained by Sanjak et al. (2010), showing that 8 weeks of repetitive rhythmic exercise mediated by supported treadmill ambulation training (STAT) for 30 min, 3 days a week, had positive effects on the ALS Functional Rating Scale (ALSFRS), distance and length of the step during 6 min of walking tests in ALS patients, confirming the beneficial role of physical exercise. Clawson et al. (2018) demonstrated that a fitness program of 24 weeks with resistance exercises using a cyclette did not promote any effect on the muscle strength and functionality and on the quality of life. Furthermore, a study conducted by Lunetta et al. (2016) showed that an active and/or passive exercise program (twice weekly) applied to the four limbs did not produce positive effects on the ALSFRS score, but it induced an improvement in the mood of ALS patients (Lunetta et al., 2016).

On the other hand, a resistance exercise program can be considered a good non-pharmacological adjuvant therapeutic approach in ALS, improving muscle force, inducing muscle hypertrophy, and maintaining skeletal muscle function (Tsitkanou et al., 2019). A randomized controlled trial in ALS patients showed that a moderate resistance training (6 months) can induce significantly better function, less decline in leg strength, and higher quality of life without adverse effects (Bello-Haas et al., 2007).

No conclusive data regarding the protective or detrimental role of physical activity in ALS population are available because of limited number of trials with low sample size and the difficulty to apply a standardized exercise protocol because of the heterogeneous progression rate of the disease clinical course.

#### Peripheral Neuropathies

Conceptually, oxidative stress in PN not only can be part, as for the other NMD, of the underlying pathogenic mechanism but also can be an expression of it and may, conversely, modulate an important aspect of PN, the reinnervation process. This aspect can accompany at least those diseases in which treatment is able to slow down or remit the disease process. Experimental studies in mice model of olfactory degeneration show how acute *N*-acetylcysteine (NAC) administration is able to ameliorate loss of olfactory neurons *in vivo* and, in an olfactory cell culture model, to alter the expression of several genes involved in oxidative stress pathways (Goncalves and Goldstein, 2019), suggesting a putative therapeutic effect of antioxidant compounds.

This also can happen by the involvement of nitric oxide (NO) and nitric oxide synthases (NOS) system, as shown in experimental models of denervation and reinnervation in rats. In his paper, the author (Tews, 2001) discussed how downregulation of sarcolemmal motor neuronal NOS in denervated muscle may contribute to axonal regeneration and attraction to muscle fibers by reducing the detrimental effects of NO nerve fiber growth. These mechanisms, therefore, stimulate the formation of new motor endplates for reinnervation process. However, decreased NO production in denervation process reduces the scavenger function of NO, promoting oxidative stress by the increase in O_2_⋅−.

Free radicals cause oxidative damage that induces axonal degeneration and segmental demyelination.

Apart from diabetic neuropathy (DN), in which pathophysiological mechanisms due to hyperglycemia are well known and characterized by increase in oxidative stress through different metabolic pathways, in other types of PN, data are still lacking. In particular, polyol metabolism increases the formation of advanced glycation end products (AGEs), the activation of protein kinase C (PKC), and the hexosamine pathway, which are primarily linked to the development of DN.

In some inherited neuropathies, such as Charcot-Marie-Tooth-2K (CMT2K), p.C240Y mutation in GDAP1-GST is associated to a mitochondrial complex I defect with a greater oxidative stress (Cassereau et al., 2020).

Nevertheless, in inflammatory peripheral neuropathies, the presence of ROS is known to be a central feature in the processes of myelin rearrangement for immune reactions. In fact, the therapeutic effect of intravenous immunoglobulins (IVIg) is related to the modulation of ROS activity on removal of the autoreactive T cells by NOX pathway (Marrali et al., 2016).

In Guillain–Barré syndrome (GBS), Tang et al. (2017) found that redox status was altered, as demonstrated by decreased levels of lipophilic antioxidant defenses, mainly γ-tocopherol and δ-tocopherol concentrations in plasma of patients. In addition, also lower serum uric acid and albumin levels, which are markers that correlate with oxidative stress, were found to be decreased in patients than in healthy controls (Su et al., 2017). Based on our knowledge, studies on the role of physical training in GBS are still lacking.

Regarding chemotherapy-induced peripheral neuropathy, some antitumoral agents, such as oxaliplatin, induce oxidative stress reactions. These mechanisms, on the one hand, lead to cancer’s cell death; on the other hand, it is well known to lead to neurotoxicity (Laurent et al., 2005).

It has been demonstrated that physical exercise, including combined exercise protocols of endurance, strength, and sensorimotor training, in cancer patients suffering peripheral neuropathy improves physical functions (Duregon et al., 2018), but no data are available regarding the possible relation with oxidative stress. Similarly, it is not possible to establish in a conclusive manner if physical exercise could counteract the detrimental effect of oxidative stress in the natural course of other different pathogenic forms of neuropathies.

## Conclusion

An imbalance between ROS production and antioxidant capacity can be a consequence of a sedentary lifestyle on the one hand and an excessive physical activity on the other hand, both in healthy people and in patients with NMD.

To improve motor functions, muscle weakness, and fatigue, targeted training protocols, which should take into consideration the planning type, workload, and session duration of the exercise, should be part of the motor regimen that can be advised within the different pathological frames of NMDs. Considering the expected natural history of each disease, aerobic and low-intensity exercise, due to its capacity to limit the detrimental effect of oxidative stress, could be considered, for different reasons, a potential non-pharmacological approach to induce beneficial adaptations in the neuromuscular system. It promotes muscle endurance, muscle strength/power, muscle trophism sparing or growth, and physical and respiratory functions in several forms of NMDs, improving the quality of life in NMD patients. Further studies are, however, necessary to optimize these therapeutic strategies in the setting of a neuromuscular disorder, likely supported by the use of reliable biomarkers, in order to better understand the context within which and at which extent the single disease mechanism can interact with tissue redox signaling, also in view of targeting their possible focused therapeutic interventions.

## Author Contributions

GS contributed to the planning and assembly of the manuscript. LC contributed to the writing and bibliography review for the chapter on muscle diseases. AL contributed to the writing and bibliography review for the chapter on ALS. CS contributed to the manuscript criticism. ES contributed to the writing and bibliography review for the chapter on peripheral neuropathies. GR contributed to the revision.

## Conflict of Interest

The authors declare that the research was conducted in the absence of any commercial or financial relationships that could be construed as a potential conflict of interest.
